# An intronic alteration of the fibroblast growth factor 10 gene causing ALSG-(aplasia of lacrimal and salivary glands) syndrome

**DOI:** 10.1186/1471-2350-9-114

**Published:** 2008-12-22

**Authors:** Kathrin Scheckenbach, Vera Balz, Martin Wagenmann, Thomas K Hoffmann

**Affiliations:** 1 Department of Otorhinolaryngology, Heinrich-Heine-University, Düsseldorf, Germany

## Abstract

**Background:**

A combined aplasia, hypoplasia or atresia of lacrimal points and salivary glands is rarely diagnosed. Those patients suffer from epiphora, xerostomia and severe dental caries. This phenotype represents the autosomal-dominant aplasia of lacrimal and salivary glands syndrome (ALSG). Recently, aberrations of the *Fibroblast Growth Factor 10 *(*FGF10*) gene have been identified to be causative for this disorder.

**Methods:**

We performed a sequence analysis of the *FGF10 *gene of a patient with ALSG-syndrome and his also affected brother as well as 193 controls. The FGF10 transcript was analyzed using RNA extracted from primary fibroblasts of the patient's mucosa.

**Results:**

We detected a novel heterozygous sequence variation in intron 2 (c.430-1, G > A) causing the ALSG syndrome. The alteration derogates the regular splice acceptor site and leads to the use of a new splice acceptor site 127 bp upstream of exon 3. The aberration was detected in the genomic DNA derived from two affected brothers, but not in 193 control individuals. Furthermore, no diseased member of the family displayed additional abnormalities that are indicative for the clinically overlapping lacrimo-auriculo-dento-digital syndrome (LADD).

**Conclusion:**

This family-based approach revealed an intronic variation of the *FGF10 *gene causing ALSG-syndrome. Our results expand the mutational and clinical spectrum of the ALSG syndrome.

## Background

A combined aplasia, hypoplasia or atresia of lacrimal points and salivary glands is rarely diagnosed. Affected patients suffer from irritable eyes, epiphora (constant tearing) and xerostomia (dryness of the mouth), accompanied with an increased risk of dental erosion, dental caries, periodontal disease, and oral infections. Those findings were described for the first time in the context of the LADD syndrome (OMIM #149730), (also known as Levy-Hollister syndrome) in 1967 [1]. In contrast to the ALSG syndrome (OMIM #180920), it displays a more severe phenotype and is characterized additionally by anomalies of the face, teeth, digits, toes, and ears including hearing loss, and hypospadias. Both sporadic and familial cases have been described for both syndromes.

Recently, genes involved in the FGF signaling pathway were associated with both syndromes. Sequence alterations in the intracellular tyrosine-kinase domains of *FGFR2 *and *FGFR3 *were described to be involved in LADD syndrome [2,3], and aberrations of the *FGF10 *gene were found to be associated with both the ALSG and the LADD syndrome [2-6]. In the present study we describe the clinical appearance of a patient that presented with an aplasia of both lacrimal punctae and bilateral aplasia of both parotid glands and the associated excretory ducts (ALSG syndrome). Various members of his family were affected suggesting a hereditary autosomal-dominant disorder. These findings prompted us to perform genetic analysis to identify the pathogenic sequence alteration underlying the mild phenotype of the ALSG syndrome in the German family.

## Methods

### Patients and controls

A 34-year old, Caucasian male presented with persisting epiphora since birth, dry mucosa of the mouth and actually a massive mucocele of the right lacrimal sac. His brother displayed, except for the lacrimal mucoceles, the same symptoms. Furthermore, the patient's father, grandmother, and grandaunt were also known to suffer from epiphora, bad dental status and xerostomia. However, these relatives are either deceased or were not willing to participate in our study.

The control group comprised 193 healthy individuals (blood donors). Patient and controls were matched with regard to ethnicity (white Caucasians) and residence (Germany).

Research was carried out in compliance with the Helsinki Declaration. This study was presented to the ethics committee of the University of Düsseldorf and biopsies and blood samples were obtained after informed consent.

### Sequence analysis of the *FGF10 *gene

For sequence analysis of the *FGF10 *gene, blood samples from both patient and his brother, and the control group were obtained, and genomic DNA (gDNA) was extracted from PBMCs using the QIAamp Blood Kit according to the manufacturer's protocols (Qiagen, Hilden, Germany). Quality of DNA was controlled by PCR of the beta-actin gene. For the patient and his brother, the entire three exons including the 5'-UTR and the 3'-UTR and flanking intronic sequences of the *FGF10 *gene were amplified applying a standard PCR protocol. PCR products were purified, and sequence analyzed in both directions using the BigDye v3.1 chemistry and the 310 Prism Genetic Analysis system (Applied Biosystems, Weiterstadt, Germany). Sequences obtained were screened for the presence of sequence variations by alignment analysis with the reference sequence derived from the GenBank database using the Sequence Navigator software (Applied Biosystems). The identified aberration was verified by repeated sequence analysis from a new amplified PCR product.

The frequency of the detected aberration was estimated in the control cohort. All primer sequences, amplicon sizes, and amplification conditions are given in Table 1.

**Table 1:** Oligonucleotide primers for amplification and sequence analysis of FGF10 in transcript and genomic DNA, and quality controls.

**Fragment**	**Tag**	**Sequence**
**Sequence analysis of the FGF 10 gene (gDNA)**		

5'-UTR	5-UTR-1	5'-CTC TCT CCT TGC CTT GCA TC-3'
Amplicon size: 456 bp	1–2	5'-CTG GGG GTG GAT AAT TGG A-3'

Exon 1	1-1	5'-CAA TTT CCA GTG CCG AGA GT-3'
Amplicon size: 400 bp	1–2	5'-CTG GGG GTG GAT AAT TGG A-3'

Exon 2	2-1	5'-GGA AAT ACT TGC CGG GTT TT-3'
Amplicon size: 439 bp	2-2	5'-GCC CTT GAA ACA GTG GTG AC-3'

Exon 3	3-1	5'-GGA GTT CTC TGG ATG GCT TG-3'
Amplicon size: 392 bp	3-2	5'-AAG CAG ACA TCT GCA ACG TG-3'

3'-UTR	UTRS	5'-GGA GTT CTC TGG ATG GCT TG-3'
Amplicon size: 1008 bp	PD3	5'-ATG CAT GTG GTG TGC TGA AT-3'

Amplification conditions:		
1 × 3 min at 94°C//35 × 30 sec at 94°C, 30 sec at 60°C, 1 min at 72°C//1 × 15 min at 72°C		

**Sequence analysis of the FGF10 transcript (cDNA)**		

5'-UTR	5-UTR-1	5'-CTC TCT CCT TGC CTT GCA TC-3'
Amplicon size: 330 bp	PD1	5'-TAG CTT TCT CCA GCG GAC AT-3'

Translated region		
(exons 1–3)	1-1	5'-CAA TTT CCA GTG CCG AGA GT-3'
Amplicon size: 770 bp	3-2	5'-AAG CAG ACA TCT GCA ACG TG-3'

Amplification conditions:		
1 × 3 min at 94°C//35 × 20 sec at 94°C, 20 sec at 60°C, 1 min at 72°C//1 × 7 min at 72°C		

3'-UTR	PU2	5'-TCG GAG TTG TTG CCG TCA AAG-3'
Amplicon size: 1005 bp	PD3	5'-ATG CAT GTG GTG TGC TGA AT-3'

Amplification conditions:		
1 × 3 min at 94°C//35 × 20 sec at 94°C, 20 sec at 63°C, 1 min at 72°C//1 × 7 min at 72°C		

**Analysis of alternative transcripts**		

Long range PCR		
(Exons 2–3)	PU2	5'-TCG GAG TTG TTG CCG TCA AAG-3'
Amplicon size:	PD3	5'-ATG CAT GTG GTG TGC TGA AT-3'
gDNA = 6239 bp		
cDNA wild type = 1005 bp		

Amplification conditions:		
1 × 2 min at 93°C//10 × 15 sec at 93°C, 15 sec at 65°C, 8 min at 68°C//23 × 15 sec at 93°C, 15 sec at 63°C, 8 min + 20 sec elongation per cycle at 68°C		

RT-PCR for polyacrylamid gels (semi-quantitative RT-PCR)		

FGF10 Exons 1–3:	E1	5'-CGG GAA GGT CAG CGG GAC CA-3'
Amplicon size: 227 bp	E3as	5'-TGC TGC CAG TTA AAT GAT GC-3'

FGF10 Exons 2–3:	PU2	5'-TCG GAG TTG TTG CCG TCA AAG-3'
Amplicon size: 416 bp	E3, 3'-UTR	5'-CCT TCA AAG GCT GGC TTT CT-3'

beta-2-microglobulin	B2Ms	5'-TTC ATC CAT CCG ACA TTG AA-3'
Amplicon size: 465 bp	B2Mas	5'-TCT CTG CTC CCC ACC TCT AA-3'

Amplification conditions:		
1 × 15 min at 95°C//40 × 30 sec at 93°C, 90 sec at 60°C, 1 min at 72°C//15 min at 72°C		
(aberrant transcript detection)		
1 × 15 min at 95°C//32 × 30 sec at 93°C, 90 sec at 60°C, 1 min at 72°C//15 min at 72°C		

**Analysis of the FGF10 transcript stability (cDNA, real time RT-PCR)**		

Exon 1/2	P1	5'-CTC CTT CTC CTC TCC TT-3'
Amplicon size: 179 bp	P2	5'-GAT GTT ATC TCC AGG ATG CTG TA-3'
	Probe E1/2	5'-GCT ATT CTC TTT CAC C-3'

Exon 2/3	P3	5'-GGG AAA CTC TAT GGC TCA AAA-3'
Amplicon size: 110 bp	P4	5'-CCT CCC ATT ATG CTG CCA GTT A-3'
	Probe E2/3	5'-GGA AAA TGG ATA CAA TAC-3'

Amplification conditions:		
1 × 15 min at 95°C//35 × 15 sec at 94°C, 30 sec at 56°C, 30 sec at 76°C		

**Control amplifications**		

GAPDH (cDNA)	GA1	5'-ACC ACA GTC CAT GCC ATC AC-3'
Amplicon size: 451 bp	GA2	5'-TCC ACC ACC CTG TTG CTG TA-3'

Amplification conditions:		
1 × 3 min at 94°C//25 × 20 sec at 94°C, 20 sec at 66°C, 30 sec at 72°C//1 × 7 min at 72°C		

beta-actin (gDNA)	BA1	5'-AGG CCA ACC GCG AGA AGA TGA-3'
Amplicon size: 732 bp	BA2	5'-GAA GTC CAG GGC GAC GTA GCA-3'

Amplification conditions:		
1 × 3 min at 94°C//30 × 20 sec at 94°C, 20 sec at 60°C, 30 sec at 72°C//1 × 7 min at 72°C		

### Analysis of the *FGF10 *transcript

#### Culture of primary fibroblasts

FGF10 is known to be expressed in fibroblasts [7]. Therefore, cell cultures of primary fibroblasts deriving from biopsies of the mucosa from the patient and three healthy individuals were established. Briefly, small biopsies were obtained from oral mucosa and placed into plastic flasks (Becton Dickinson, Heidelberg, Germany). Flasks were flooded with fibroblast growth medium (Cambrex Bio Science, Verviers, Belgium) and incubated under cell culture standard conditions (37°C, 5% CO_2_, fully humidified atmosphere). Fibroblasts grew out on day 3–5. To transfer or passage the fibroblasts, monolayers were washed with PBS and detached with 0.05% trypsin/0.02% EDTA solution (Biochrom). Cells were used for analysis between the third and the eighth passage.

#### Conventional RT-PCR

From each fibroblast line, total RNA was prepared on using the RNeasy Kit including an on-column DNAse digestion step according to the manufacturer's protocol (Qiagen). 5 μg of total RNA was reverse transcribed using a mixture of random 15-mer oligonucleotides and anchored oligo-dT primers and M-MLV reverse transcriptase (Sigma) in a standard protocol. Aliquots of the conversion mixture were amplified by PCR in a thermal cycler (Biometra, Göttingen, Germany) with specific primers (Table 1) and Qiagen Master Mix (Qiagen), subdividing the FGF10 transcript into 3 overlapping amplification products. The resulting PCR products were controlled using conventional agarose gel electrophoresis and sequence analyzed according to the experimental procedure as outlined for amplificates of the genomic DNA. The quality of the cDNA was controlled by RT-PCR of GAPDH.

#### Long Range RT-PCR

To analyze for aberrantly transcripts that may include the entire intron 2 of the *FGF10 *gene, we established a long range PCR procedure using gDNA from control PBMCs as template and primers that are located in exon 2 (sense) and exon 3 (antisense) ensuring that both gDNA and cDNA templates are amplified. Briefly, 200 ng gDNA were set in a reaction volume of 50 μl using 500 μM dNTP (each), 0.4 μM of each primer, and 4 units TripleMaster polymerase mix in 1 × Tuning buffer (Eppendorf AG, Hamburg, Germany) to amplify the *FGF10 *gene. Applying these conditions, aliquots of the cDNA (equivalent to 200 ng total RNA) were used to amplify the FGF10 transcript. The obtained PCR products were analyzed using conventional agarose gels stained with ethidium bromide. All primer sequences, amplicon sizes and amplification conditions are given in table 1.

#### Identification of the aberrant transcript

The transcript in question may undergo rapid nonsense-mediated mRNA decay and, thus, may not be detected using conventional agarose gels stained with ethidium bromide. Therefore, we decided to use a more sensitive separation and detection system. RT-PCR amplificates were separated on precasted polyacrylamid gels (CleanGel, Amersham Biosciences, Freiburg, Germany) according to the manufacturers' protocol and silver stained (DNA Silver Staining Kit, Amersham Biosciences). Aberrant bands were excised from the polyacrylamid. The excised fragments were placed in dH_2_O and heated for 30 min at 95°C. 1 μl of the eluted DNA was re-amplified and the resulting PCR product was sequenced.

### Algorithmic splice site prediction

To analyze the effect of the intronic alteration on mRNA splicing, we conducted automated splice site prediction using two software programs (NetGene2 and NNSplice 0.9, available at  and , respectively).

### Accession numbers

GenBank: human FGF10 transcript, NM_004465.1; human *FGF10 *gene, AY604046.1

## Results

### Clinical features

We examined a patient and his brother with clinical findings of the hereditary ALSG-syndrome.

**Figure 1:**
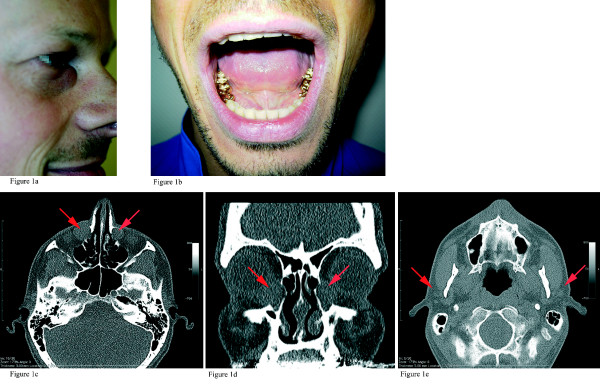
**Computer tomography scans demonstrating the pathological findings of an ALSG patient**. The pathological areas are marked. 1a: The patient shows an aplasia of both lacrimal punctae associated with a mucocele of the lacrimal sac imposing as a benign soft tissue tumor in the region of the medial canthus. 1b: Prosthetically (gold cap) treated teeth after caries-related destruction, especially in the molar region. 1c-d: Axial (c) and coronal (d) computer tomography scan demonstrating mucoceles originating from the lacrimal sac. The os lacrimale is partly destroyed, on the right side more pronounced than on the left side. 1e: Axial computer tomography scan showing a total aplasia of both parotid glands.

Clinical exploration revealed an aplasia of both lacrimal punctae associated with a mucocele of the lacrimal sac imposing as a benign soft tumor in the region of the medial right canthus and bilateral aplasia of both parotid glands and the excretory ducts (Fig. 1a). In addition, prosthetical and conservatively treated teeth after caries-related destruction, especially in the region of the molars, were observed (Fig. 1b). The mucocele which had already led to bony erosion of the os lacrimale was seen in ultrasonography as well as in the computer tomography scan (Fig. 1c–d). Those images confirmed a total aplasia of both parotid glands also, whereas both submandibular and sublingual glands were present (Fig. 1e). An endoscopic dacryocystorhinostomia interna (West operation) was performed on both sides to drain the lacrimal sacs. Clinical examination of the patient's brother who, except for the lacrimal mucocele, suffered from the same symptoms, also revealed an aplasia of both lacrimal punctae and a total aplasia of submandibular, sublingual as well as parotid glands. In addition, his teeth were treated in a prosthetical and conservative way. Anomalies of the teeth, fingers, toes, and ears including hearing loss were neither found in the patient nor in his brother. The genitourinary system was normal.

### Mutation analysis

**Figure 2:**
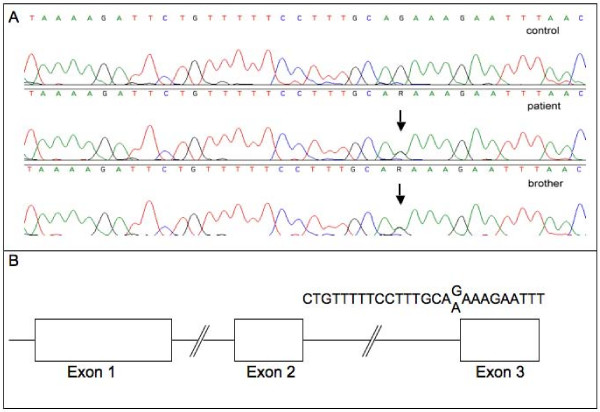
**a: A heterozygous sequence variation in the terminal nucleotide of intron 2 of the *FGF10 *gene (g.85478 corresponding to c.430-1, G > A) was detected which alters the consensus motif for splice acceptor site recognition**. The upper panel shows a representative electropherogram of a healthy individual. The middle and the lower panel demonstrate the sequence variation in genomic DNA derived from the patient and his brother. The changed nucleotide is marked by an arrow. 2b: Chart showing the exon/intron structure of the *FGF10 *gene. The characteristic polypyrimidine stretch and the consensus acceptor site are shown. The changed nucleotide is marked by an arrow.

The recently described interrelation between the ALSG syndrome and alterations in the *FGF10 *gene prompted us to perform sequence analysis of the entire three exons, the 5'- and 3'-UTRs, and exon flanking sequences of the *FGF10 *gene in gDNA derived from patient's PBMCs. Five different single nucleotide polymorphisms are described for the FGF10 transcript with variable allele frequencies, located in the 5'-UTR, in the translated part of exon 3 and in the 3'-UTR (1, 1, and 3 polymorphisms, respectively). However, none of these SNPs was found. However, we detected a heterozygous sequence variation in the terminal nucleotide of intron 2 (c.430-1, G > A) altering the consensus motif for splice site recognition (Fig. 2). The aberration was also found in gDNA isolated from PBMCs of the patient's brother who also suffers from ALSG. Moreover, it was not detected in gDNA derived from 386 control chromosomes nor reported in the GeneSnp database or in the Human Gene Mutation database available at  and , respectively.

### Effects on mRNA splicing

To assess whether the alteration affects mRNA splicing of FGF10, two different splice site prediction algorithms were used. Both algorithms identified the donor and acceptor sites of the three exons of the *FGF10 *gene correctly when provided with the wild type sequence. In contrast, after substitution of the terminal nucleotide of intron 2 the loss of the splice acceptor site of exon 3 was anticipated by both of the algorithms. Moreover, both algorithms show potential alternative splice sites in intron 2 or exon 3 which would result in the addition of parts of intron 2 or the deletion of exon 3 sequences (Table 2).

**Table 2:** Splice site prediction with NNSplice 0.9 and NetGene2 v. 2.4. AY604046.1

**NNSplice 0.9**				
**Acceptor site predictions:**				

**Start**	**End**	**Sequence (5'-> 3')**	**Function**	

80131	80150	tatgtttt**ag**gcatcctgga	Exon 2 acceptor	

85469	85488	tcctttgc**a*g***aaagaattta	Exon 2 acceptor	

				

**Alternative acceptor sites:**				

**Start**	**End**	**Sequence (5'-> 3')**	**Localization**	**Effect on FGF10 transcript**

81353	81372	tccctccc**ag**agacagatca	Intron 2	insertion of 4116 bp

81899	81918	ctaatatc**ag**gtccactttt	Intron 2	insertion of 3570 bp

82609	82628	ctcccaat**ag**attgtgacct	Intron 2	insertion of 2860 bp

84430	84449	ctattttt**ag**tatagacggg	Intron 2	insertion of 1039 bp

85062	85081	tttccttc**ag**ttctattttt	Intron 2	insertion of 407 bp

86294	86313	tctttcta**ag**ttatttattt	Exon 3, 3'-UTR	deletion of 825 bp

86384	86403	ttttattc**ag**cacaccacat	Exon 3, 3'-UTR	deletion of 915 bp

				

**NetGene2 v. 2.4**				

**Acceptor site predictions:**				

**position 5'-> 3'**		**Sequence (5'-> 3')**	**Function**	

80140		TATGTTTTAG*GCATCCTGGA	Exon 2 acceptor	

85478		TCCTTTGCAG*AAAGAATTTA	Exon 2 acceptor	

**Alternative acceptor sites:**				

**position 5'-> 3'**		**Sequence (5'-> 3')**	**Localization**	**Effect on FGF10 transcript**

82618		CTCCCAATAG*ATTGTGACCT	Intron 2	insertion of 2860 bp

86393		TTTTATTCAG*CACACCACAT	Exon 3, 3'-UTR	deletion of 915 bp

AY604046.1 Both algorithms anticipate the loss of the regular splice acceptor site of FGF10 exon 3 caused by the c.430-1, G > A aberration. Several alternative acceptor splice sites are proposed. Localization according to GenBank Accession No. AY604046.1.

In order to determine the actual effect of the c.430-1, G > A aberration on FGF10 mRNA splicing, we undertook several experimental approaches. FGF10 is known to be expressed in fibroblasts. Therefore, we established primary fibroblast cells from both the patient and three healthy individuals and analyzed the FGF10 transcript.

**Figure 3:**
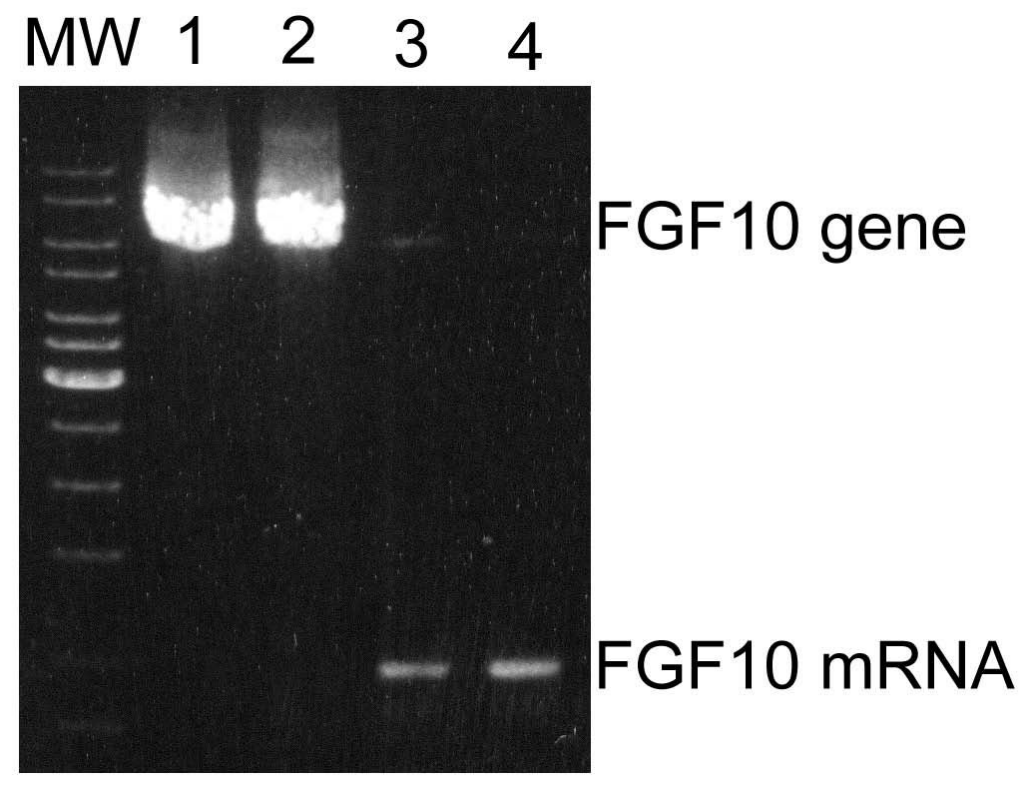
**Analysis of FGF10 transcript applying long range RT-PCR**. In order to analyze for transcripts that may include the entire intron 2, a long range RT-PCR protocol was established that works to amplify the 6.2 kb-fragment spanning from exon 2 to exon 3 using gDNA as template. Applying the optimized conditions to amplify the FGF10 transcript, no accordant PCR product was present. 1, 3: patient; 2, 4: control; MW: molecular weight marker.

To eliminate the possibility of the inclusion of the entire intron 2, which is too large to be amplified under normal PCR conditions, a long range RT-PCR protocol was established that works to amplify the 6.2 kb-fragment spanning from exon 2 to exon 3 using gDNA as template. However, applying the same protocol on the patient's cDNA no accordant PCR product was present (Fig. 3).

**Figure 4:**
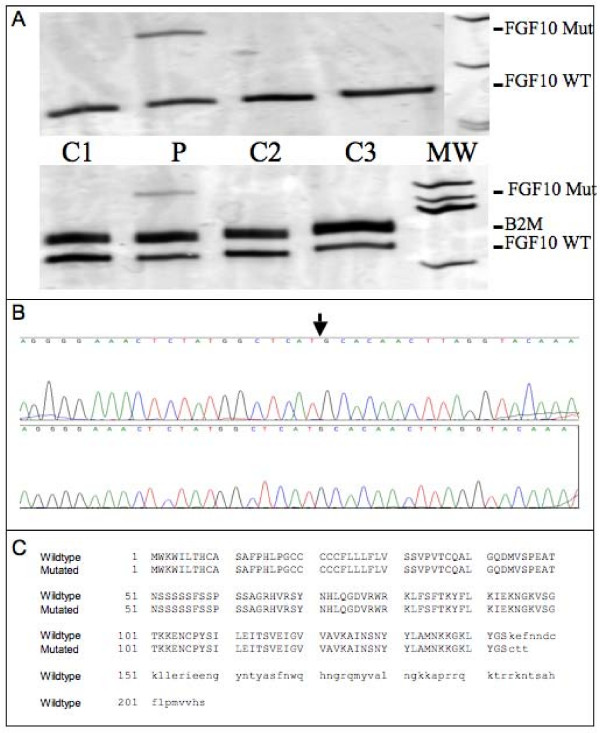
**Detection and analysis of an aberrant transcript**. 4a: Separation of FGF10 RT-PCR amplificates using sensitive silver-stained polyacrylamid gels reveals additional fragments for the patient's samples but not for control samples. Upper panel, Amplification of the FGF10 transcript using oligonucleotides designed to amplify exon 1 to exon 3 sequences; Lower panel, Amplification of the FGF10 transcript using oligonucleotides designed to amplify exon 2 to exon 3 sequences. C: control; P: patient. 4b: Sequence analysis of the excised and re-amplified additional exon 1/exon 3 fragment. The c.430-1, G > A aberration results in the use of an alternative splice acceptor site 127 bp upstream of exon 3. 4c: Putative effect of the 127 bp insertion on protein translation.

In case that one of the potential acceptor sites is used, RT-PCR amplificates of the affected and the wild type transcript deviate in molecular size. However, FGF10 mRNA amplificates from the patient did not show an anomalous molecular weight by size separation using conventional agarose gel electrophoresis. In contrast, using the more sensitive separation and detection system of silver-stained polyacrylamid gels, we were able to identify aberrant migrating fragments in RT-PCR reactions using two different primer combinations for the patient's samples but not for control samples (Fig. 4a). Re-amplification and sequence analysis of the excised DNA fragments revealed the use of an alternative acceptor splice site upstream of exon 3 that results in the insertion of additional 127 bp into the FGF10 transcript (Fig. 4b). However, this acceptor splice site has not been foretold by any of the splice site prediction algorithms. The insertion leads to a preliminary translation stop codon after the insertion of three novel amino acids following amino acid 143 (Fig. 4c).

## Discussion

Recently, Entesarian et al. [5] showed that alterations of the *FGF10 *gene are causative for the described phenotype. In each of two diseased families a heterozygous sequence variation of the *FGF10 *gene was detected and an autosomal-dominant hereditary transmission was documented. The alteration of one family represents a 53-kb deletion concerning exons 2 and 3, and the other family carried a nonsense mutation (p.R193X; c.577C > T). Because both alterations result in the loss of major parts of the FGF10 protein they are considered to be non-functional. A correlation of these *FGF10 *aberrations and sporadic cases of sicca-syndrome with symptoms identical to those of individuals with ALSG was excluded by screening DNA samples from 74 patients. Moreover, heterozygous *FGF10 *mice developed the same phenotype [5]. More recently, two missense mutations of the FGF10 gene (p.R80S; c.240A > C and p.G138E; c.413G > A) were shown to be causative for the ALSG syndrome [4]. Both of these mutations exchange highly conserved and functionally important residues suggesting the production of a non-functional protein or a protein with an altered function. In addition, the LADD syndrome is also caused by *FGF10 *mutations indicating that both syndromes are allelic disorders and part of the same phenotypic spectrum [2,6]. Here, two missense mutations in exon 1 (p.C106F; c.317G > T) and exon 3 (p.I156R; c.467T > G), and a nonsense mutation in exon 2 (p.K137X; c.409A > T) were detected.

These studies prompted us to perform genetic analysis to identify the pathogenic sequence alteration underlying the mild phenotype of the ALSG syndrome in the German family.

We detected a novel *FGF10 *sequence variation in intron 2 altering the consensus acceptor site of the terminal exon 3 (c.430-1 G > A). This may affect the resulting transcript in different ways. Due to the use of an alternative acceptor site in either intron 2 or exon 3, additional flanking intronic sequences may be inserted, or the 5'-part of exon 3 may be deleted, frequently resulting in a frameshift. In fact, we were able to find an altered transcript in patient's mucosal fibroblast cells. The *FGF10 *alteration causes the use of an alternative acceptor splice site which is located 127 bp upstream of exon 3. This mutation results in a premature translation termination codon immediately after the insertion of three novel amino acids following exon 2. As a consequence the peptide lacks a large C-terminal part of the molecule and will probably not have any biological activity because its FGF10 core will have been severely disrupted.

Transcripts containing frameshift or nonsense mutations that cause premature translation termination codons are often degraded by the nonsense-mediated mRNA decay mechanism [8]. It is known, that aberrations in the 5'-UTR or 3'-UTR may result in instability of mRNA. However, sequence analysis did not identify any further alteration. On the other hand, additional nucleotide variation in the promoter of the *FGF10 *gene could lead to diminished transcription for example through abrogation of transcription factor binding sites.

The LADD- and ALSG syndromes are allelic disorders. However, the majority of the detected *FGF10 *mutations are described to cause the milder ALSG syndrome – including ours – are predicted to result in a non-functional protein. Recent studies and our findings suggest that changed FGF10 signaling due to haploinsufficiency during development results in ALSG. In contrast, for LADD syndrome patients two *FGF10 *missense alterations were found which result in the exchange of less conserved residues suggesting residual or possibly altered activity of the aberrant protein [4].

## Conclusion

To the best of our knowledge, this is the first report of a splicing mutation in the *FGF10 *gene. The detected mutation leads to the insertion of additional intronic 127 bp upstream of the terminal exon 3. This alteration does not add up in more severe anomalies which are associated with the allelic LADD syndrome but in a mild ALSG phenotype. To date, no functional differences are described between FGF10 ALSG and LADD mutations. Future clinical and genetic investigations of additional affected families are mandatory to provide the rationale to distinguish the clinical outcome of the disruption of FGF10 signaling.

## Abbreviations

ALSG: aplasia of lacrimal and salivary gland; FGF: fibroblast growth factor; FGFR: fibroblast growth factor receptor; gDNA: genomic DNA; PBMC: peripheral blood mononuclear cells; RT-PCR: reverse transcriptase-polymerase chain reaction.

## Competing interests

The authors declare that they have no competing interests.

## Authors' contributions

KS investigated the patient, performed the sequence analysis and wrote most passages of the manuscript. VB performed the FGF-transcript analysis including the fibroblast culture, carried out real-time RT-PCR and wrote certain passages of the manuscript. MW wrote certain passages of the manuscript, improved the written English and was responsible for the photographs and graphs. TKH investigated the patient, performed surgery and reviewed the paper. All the authors read and approved the final manuscript.

## Pre-publication history

The pre-publication history for this paper can be accessed here:


